# T147. DECREASED STRIATAL REWARD PREDICTION ERROR CODING IN UNMEDICATED SCHIZOPHRENIA PATIENTS

**DOI:** 10.1093/schbul/sby016.423

**Published:** 2018-04-01

**Authors:** Teresa Katthagen, Jakob Kaminski, Andreas Heinz, Florian Schlagenhauf

**Affiliations:** 1 Charité – Universitätsmedizin Berlin; 2 Charité – Universitätsmedizin Berlin, Max Planck Institute for Human Cognitive and Brain Sciences

## Abstract

**Background:**

Reinforcement learning involves flexible adaptation towards a changing environment and is driven by dopaminergic reward prediction error (RPE; outcome (R) – expectation (Q)) signaling in the midbrain and projecting regions, such as the ventral striatum (Schultz, 1998). Schizophrenia patients show heightened dopamine levels in the striatum (Howes et al., 2012) as well as deficits in reinforcement learning (Waltz, 2016) which may be mediated by disrupted prediction error signaling (Heinz and Schlagenhauf, 2010; Schlagenhauf et al., 2014). Using model-based fMRI, the present study aims to assess these neural signals during a reversal learning paradigm in unmedicated schizophrenia patients and healthy individuals.

**Methods:**

In the current study, 19 schizophrenia patients and 23 age- and gender-matched healthy controls completed a reversal learning paradigm (Boehme et al., 2015) during fMRI scanning where subjects had to choose between two neutral stimuli to maximize their reward. A Rescorla Wagner learning model (Single Update, one learning rate) was fitted against the individual choice data using a softmax function. Individual RPE trajectories from the fitted Rescorla Wager learning model were correlated with the BOLD response during feedback onset. Parameter estimates of ventral striaral RPE trajectories were correlated with psychopathology scores from the PANSS (Kay et al., 1987).

**Results:**

In the reversal learning task, schizophrenia patients chose the correct stimulus less often compared to healthy individuals (percent correct choices: 65.7 ± 10.7 vs. 76.7 ± 7.7; t=3.7, p=0.001). Across all participants, the RPE trajectories correlated with BOLD response in the bilateral ventral striatum (left ventral striatum [-10 12 10], t=7.40, pFWE <0.001, right ventral striatum [10 12 -10], t=6.56, pFWE=0.006). Schizophrenia patients displayed decreased RPE coding in the right ventral striatum compared to healthy individuals ([14 14 -10], t=3.69, pSVC for nucleus accumbens = .015). In patients, extracted parameter estimates from the right ventral striatum correlated negatively with the PANSS total symptoms score (Spearman’s rho =-0.55, p=0.018).

**Discussion:**

We found that unmedicated schizophrenia patients performed worse in the reversal learning task and displayed decreased striatal prediction error signaling. This neural deficit was increased in patients with overall higher symptom severity. While RPE coding seems to be intact in patients receiving antipsychotic medication (Culbreth et al., 2016), our findings are in line with previous studies in unmedicated schizophrenia patients (Reinen et al., 2016; Schlagenhauf et al., 2014). Therefore, deficient neural coding of this core reinforcement learning mechanism may reflect a characteristic of the disorder of schizophrenia and does not result from antipsychotic medication.

